# The Role of Mobile Technologies in Health Care Processes: The Case of Cancer Supportive Care

**DOI:** 10.2196/jmir.3757

**Published:** 2015-02-12

**Authors:** Greta Nasi, Maria Cucciniello, Claudia Guerrazzi

**Affiliations:** ^1^Department of Policy Analysis and Public ManagementBocconi UniversityMilanoItaly; ^2^SDA Bocconi School of ManagementMilanoItaly; ^3^Center for Research in Health and Social Care Management (CeRGAS)Bocconi UniversityMilanoItaly; ^4^Department of Health Services AdministrationSchool of Health ProfessionsUniversity of Alabama at BirminghamBirmingham, ALUnited States

**Keywords:** mhealth, cancer supportive care, cancer care, new models of care, integrated care, health care process, care delivery value chain

## Abstract

**Background:**

Health care systems are gradually moving toward new models of care based on integrated care processes shared by different care givers and on an empowered role of the patient. Mobile technologies are assuming an emerging role in this scenario. This is particularly true in care processes where the patient has a particularly enhanced role, as is the case of cancer supportive care.

**Objective:**

This paper aims to review existing studies on the actual role and use of mobile technology during the different stages of care processes, with particular reference to cancer supportive care.

**Methods:**

We carried out a review of literature with the aim of identifying studies related to the use of mHealth in cancer care and cancer supportive care. The final sample size consists of 106 records.

**Results:**

There is scant literature concerning the use of mHealth in cancer supportive care. Looking more generally at cancer care, we found that mHealth is mainly used for self-management activities carried out by patients. The main tools used are mobile devices like mobile phones and tablets, but remote monitoring devices also play an important role. Text messaging technologies (short message service, SMS) have a minor role, with the exception of middle income countries where text messaging plays a major role. Telehealth technologies are still rarely used in cancer care processes. If we look at the different stages of health care processes, we can see that mHealth is mainly used during the treatment of patients, especially for self-management activities. It is also used for prevention and diagnosis, although to a lesser extent, whereas it appears rarely used for decision-making and follow-up activities.

**Conclusions:**

Since mHealth seems to be employed only for limited uses and during limited phases of the care process, it is unlikely that it can really contribute to the creation of new care models. This under-utilization may depend on many issues, including the need for it to be embedded into broader information systems. If the purpose of introducing mHealth is to promote the adoption of integrated care models, using mHealth should not be limited to some activities or to some phases of the health care process. Instead, there should be a higher degree of pervasiveness at all stages and in all health care delivery activities.

##  Introduction

Nowadays, health care systems are facing multiple challenges that are gradually leading to the adoption of new care models. The majority of these new care models are based on a shift away from mostly large general hospitals with fewer hospital beds dedicated to acute care and toward the delivery of more health care services in primary care settings, day care facilities, and health centers [1].

This is also true for cancer care, especially for the treatment of its side effects, known as cancer supportive care whose intention is to give patients relief from side effects such as nausea, pain, and fatigue. More precisely, the main purpose of cancer supportive care is not to cure cancer, but to manage the symptoms of cancer. To this extent, cancer supportive care is part of the treatment phase of the health care process, as it is usually given alongside the actual cancer treatment [2].

New care models put greater emphasis on the role of the patient [3] and are moving toward activities carried out by the patient on a self-management basis. More specifically, patients are required to self-manage the side effects of the care processes they are receiving. On the other hand, there is great emphasis on the effectiveness of care and on the quality of life. However, the combination of these two trends points to a tradeoff between rising costs and enhancing quality [4] and technology can play a major role in the management of this tradeoff [5].

In light of these challenges, it is important to identify the promise held by mHealth for achieving new care models, as outlined by decision makers, communications media, and literature.

According to literature, mHealth has a crucial role to play since it can improve communication and enhance the integration of care processes [6,7]. Looking at the internal processes in use at health care organizations, mHealth can increase the productivity of health care providers, and consequently may even improve the productivity of health care systems [8-11]. Focusing on the external relations of health care organizations, mHealth can enhance transparency [12,13] and so increase the accountability of health care providers and systems, but it can also empower patients [14-16]. Finally, the greatest promise of mHealth is to enhance the quality of life and the appropriateness of care [17-19].

Therefore, mHealth can help in the pursuit of new health care models, requiring a shift from inpatient to outpatient care*,* also enabling the delivery of care in rural settings and other places where there is no ready access to medical personnel [20]. More precisely, mobile phone-based initiatives can solve several of the major problems encountered in low-income countries: distance, limited computer access, and a lack of health care workers, thus enabling improvements in terms of efficiency and lower health care delivery costs [21].

mHealth appears to complement current transitions within health care models, shifting care from the acute hospital setting to the home, with technology being used to rationalize and integrate services, where appropriate, based on the patient’s needs. Moreover, mHealth can play a significant role in empowering patients, giving them the tools to manage their condition and any associated side effects themselves, in their own home and without the need for direct supervision by health care personnel [22].

This paper aims to review existing studies on the actual role of mobile technology during the different stages of care processes and how and why it is used, with particular reference to cancer supportive care. This will enable us to determine whether using mHealth actually supports the introduction of new models of care.

The systematic use of technology in health care can be traced back to the more comprehensive evolution of information systems with the gradual automation of structured, semi-structured, and unstructured processes and activities [23,24]. As a result, it is important to determine the types of data and activities that need to be designed and performed, because identifying them helps to determine the best technologies to be implemented.

We should note that mHealth is a broad concept including various types of mobile technologies. It often refers to consumer health care technologies, such as Web-based information resources, telephone messaging (short message service/SMS, multimedia messaging service/MMS), remote monitoring of patients, remote interpretation of medical reports, videoconferencing, and telehealth, including the remote services of a surgeon operating at a distance, and telerobotics [25].

More specifically, the World Health Organization [26] has stated that mHealth includes technologies like mobile phones, personal digital assistants (PDAs), and smartphones, patient monitoring devices, mobile telemedicine/telecare devices, MP3 players for mLearning, and mobile computing. Based on this classification, the category of “SMS” (or text messaging) should be kept separate from the broader description of “mobile devices”, which will be used to classify smartphones, tablets, and apps. The difference is based on the distinctive features of the two categories: SMS is a tool to remind patients of an appointment whereas a “mobile device” is an instrument that is useful for collecting and processing data. This consideration is also valid when referring to the differences between “mobile devices” and “mobile telemedicine/telecare devices”. Even if integrated with a mobile phone, telemedicine devices are standalone technologies [26] taking advantage of wireless telecommunications infrastructures and are defined as “the use of telecommunications and computer technologies, including patient remote sensing and monitoring, and the use of telemetry devices, with medical expertise to facilitate health care delivery” [8].

Mobile technology should be introduced in line with the activities it aims to support. It first supports automation [27], data collection [10,28], and then operations. However, it can also support clinical decision making [29], especially monitoring (eg, pain monitoring) [30], and the planning of activities. However, most strategic documents on mHealth issued by international organizations and leading organizations in the field, and adopted by decision makers, suggest that mHealth should assist human-executed processes and should play a fundamental role in new models of care [31,32].

If we focus on health care processes, we can examine the potential role of mHealth in the value chain of care delivery [5]. mHealth can play a role in all phases of the care delivery process, supporting prevention, diagnosis, decision, treatment, and follow-up. Since it can support data collection, monitoring, and new care models, it can contribute to the creation of value if it is embedded into the entire care process, making a difference in the way care is delivered and shifting its focus onto homecare and mobile care.

mHealth can be introduced at each phase of the health care delivery process in order to support structured activities, such as data collection, semi-structured activities like monitoring, and unstructured activities, like assisting human-executed processes.

The prevention phase uses mobile apps for promoting healthy habits by scheduling reminders, as well as more unstructured campaigns that use mobile technologies for mLearning activities aimed at teaching people about diseases.

In the diagnosis phase, mobile technology can facilitate remote access to patient information, but it can also help to carry out more complex and human-executed processes like telediagnosis. Once the diagnosis has been carried out, the clinician has to make decisions and mHealth can be helpful in several ways for decision making—from automated mobile libraries with clinical descriptions of diseases to the use of mobile technologies for shared decision making by health care professionals.

During treatment, mobile technology can be used to manage a patient’s symptoms and condition or to enable the patient to do this himself (self-management), but it can also be helpful for treating patients at remote locations by means of telehealth and telesurgery equipment.

Finally, after a patient has been treated, fundamental follow-up activities have to be put in place and these can be supported by mobile technology, for example, the real-time measuring of a patient’s vital signs or for achieving better and ongoing quality communication between patients and health care professionals. Some authors consider the follow-up and “survivorship phase” as being strictly connected. The survivorship phase includes several components, ranging from the prevention of recurrence or new cancer to the treatment of the consequences of cancer, including deferred psychological effects [33]. As the US Institute of Medicine recommends, survivorship care plans should be provided to patients at the end of their treatment in order to improve health-related outcomes such as distress, self-efficacy, and quality of life [34].

**Figure 1:**
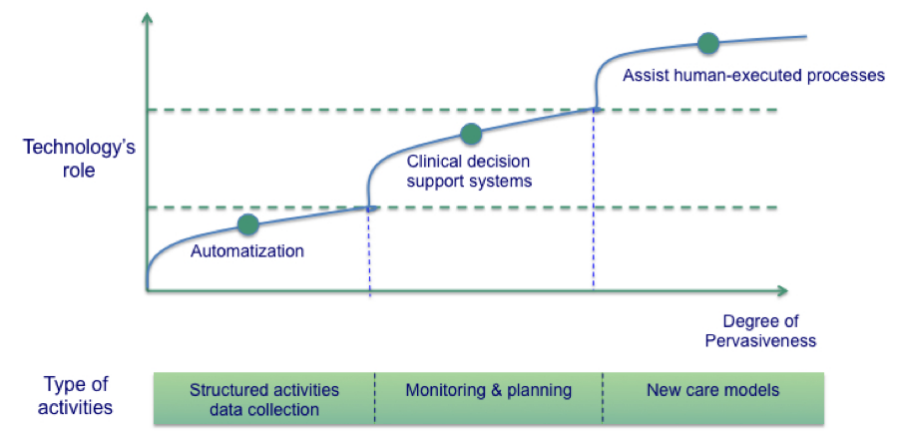
The role of mHealth.

**Figure 2:**
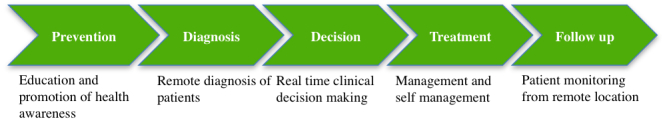
Mobile technologies in the health care process.

mHealth has the potential to make a difference in terms of better quality of life, more appropriate care, and less burden on health care processes, if it is used in its multiple roles, as shown in Figure 1, throughout the care process, as shown in Figure 2, if it is embedded in the organization or in the environment where the health care process takes place, and if it is pervasive in human executed activities.

##  Methods

**Figure 3:**
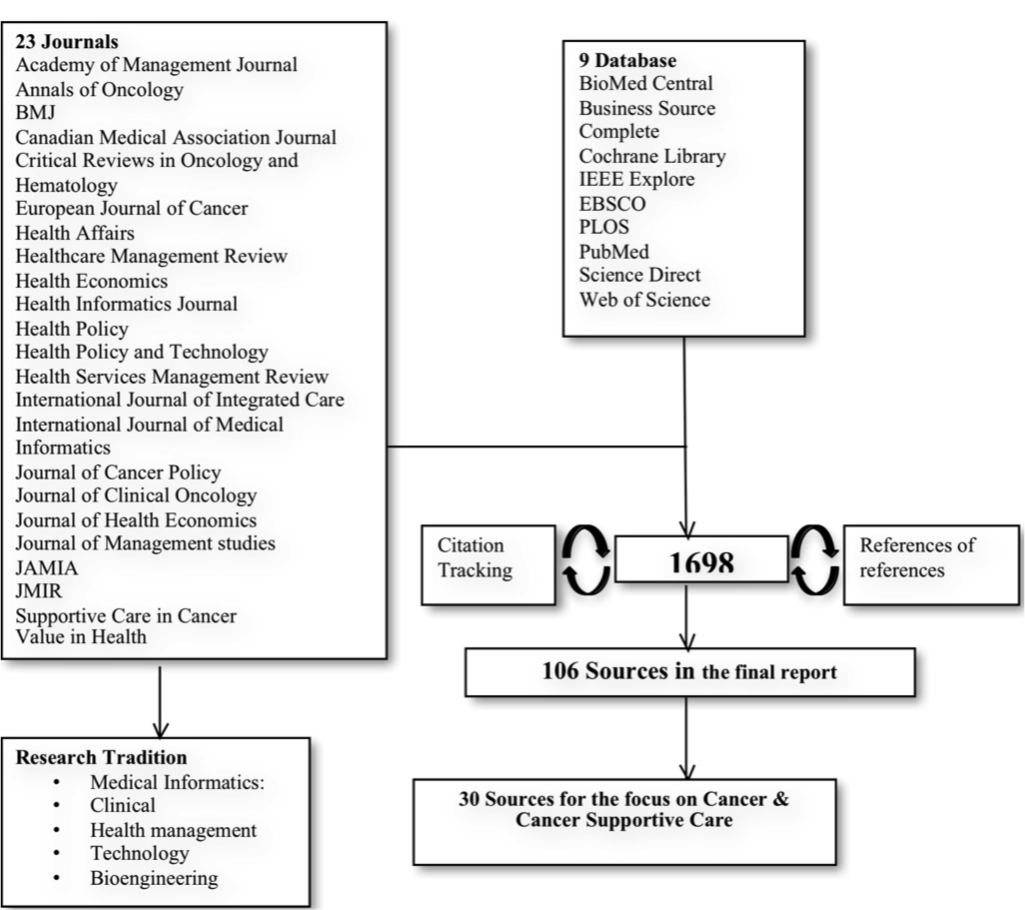
Research strategy: results.

We undertook a review of literature in order to understand the evidence relating to the actual use and role of mHealth, particularly with regard to cancer supportive care. We reviewed papers from three bodies of literature: Medical Informatics, Healthcare Management*,* and Medicine, with particular reference to oncology journals. The first step of our research strategy (Table 1, Figure 3) was aimed at identifying and collecting all existing studies of mHealth and integrated care focusing on cancer and cancer supportive care.

We then applied a “snowball” method and tracked the articles whose list of references included the works we considered fundamental for our research. We retrieved papers and studies that were published after 1999 in scholarly reviews and journals that were not listed in the database at the time of the analysis, but that were familiar to scholars. We also examined papers published in *JAMIA, JMIR, BMJ, Health Affairs, HealthCare Management Review, Health Policy,* and *Health Policy and Technology*.

**Table 1:** Research strategy.

Keywords	Generic search using the concept words: “mHealth”, “cancer”, “quality of life”
	Specific searches: “mHealth” (“mHealth” OR “mHealth” OR “mobile health” OR “mobile healthcare”) + “cancer” (cancer OR “cancer care” OR “cancer supportive care” OR “supportive care in cancer” OR “chemotherapy” OR “side effects” OR “adverse effects” OR “integrated care” OR “cancer integrated care”) “Quality of life” (“quality of life” OR “quality of service” OR “quality of care” OR “healthcare delivery” OR “healthcare management” OR “care management” OR “health policy” OR “promises” OR “continuity of care” OR “lean healthcare” OR “lean health care” OR “lean thinking” OR “patient-centered”) + “performance” (“performance” OR “evaluation” OR “impact” OR “assessment” OR “return” OR “promises” OR “adoption”)
Databases	BioMed Central, Business Source Complete, IEEE Xplore, PLOS (One, Medicine and Clinical Trials), PubMed, Science Direct, Web of Science (which embeds Elsevier, Wiley, JMIR, JAMIA), Cochrane Library
Specific Journals	JAMIA, JMIR, BMJ, Health affairs, HealthCare management review, Health Policy, Health Policy and Technology, Value in Health (ISPOR), Journal of Cancer Policy, Academy of Management Journal, Journal of Management studies, Journal of Health Economics, Health economics, Canadian Medical Association Journal, Health Informatics Journal, Journal of Clinical Oncology (ASCO), Annals of Oncology (ESMO), Supportive Care in Cancer (MASCC), European Journal of Cancer (published by Elsevier, official journal of EORTC, ECCO, EACR and EUSOMA), Critical Reviews in Oncology and Hematology (ESO), Health Services Management Review (EHMA), IEEE Antennas and Propagation Magazine, Current Oncology
Inclusion criteria	Peer reviewed published articles
	Published since 1999
Exclusion criteria	Grey literature (blogs, newsletters, videos)
	Provisional or structured abstracts
	Poster sessions, presentations, comments, opinions, discussions, editorials, prefaces, summaries, interviews, correspondence, tutorials
	Studies on psychology, ie, behavioral models and theory of psychology
	Studies where mobile health means mobile clinics or mobility of professionals or mobile screening units
	Studies or articles with no author
	Studies or articles with no abstract

##  Results

### Overview of Findings

This section describes the results of our review of existing studies on the actual role of mobile technology at the different stages of the care process.

**Figure 4:**
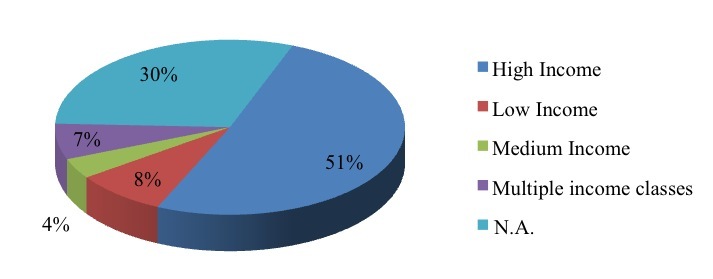
Type of country.

The first finding to be highlighted is that studies mainly refer to high income countries (50.9%, 54/106) [35,36] and focus less on low income (8.5%, 9/106) [37] and middle income countries (3.8%, 4/106) [38]. We should mention that 6.6% (7/106) of papers refer to different types of countries. A total of 30.2% (32/106) [14,29] of the selected studies do not refer to any specific country or region (Figure 4), as they review literature or describe a specific mobile technology.

**Figure 5:**
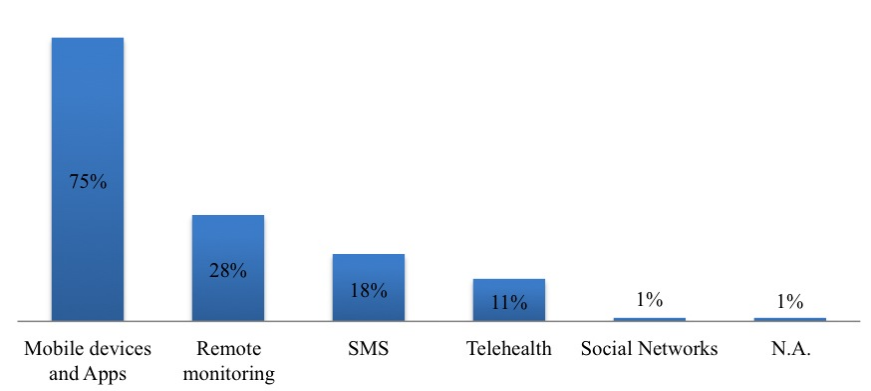
Mobile technologies.

Looking at the analysis in greater detail, we examined the role of mobile technology in health care delivery. As mentioned above, mHealth can be used for supporting structured, semi-structured, and unstructured activities, and different technologies can be introduced as a result. In particular, with regard to the type of technologies analyzed, our research found that mobile devices (like smartphones and tablets) and apps are analyzed by 75.5% (80/106) [14,17,35,36,39,40] of papers, remote monitoring technologies by 28.3% (30/106) [37] of papers, and text messaging technologies by 17.9% (19/106) [36,41] of papers (Figure 5). It should be noted that some papers refer to several types of mobile technologies. We found that mHealth is mainly used for supporting data collection, monitoring, and pain management [35,42-44], especially in cancer supportive care.

**Figure 6:**
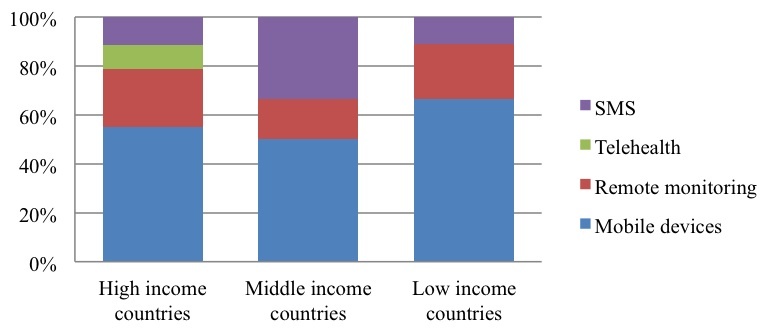
The role of mHealth in high, middle, and low income countries.

These various technologies are not spread evenly across all areas of the world: more complex processes and human-executed activities seem to be more common in high income countries. This difference can be observed in the technologies adopted; telehealth technologies are only found in high income countries for instance [45], whereas text messaging prevails in middle income countries [38] (Figure 6).

**Figure 7:**
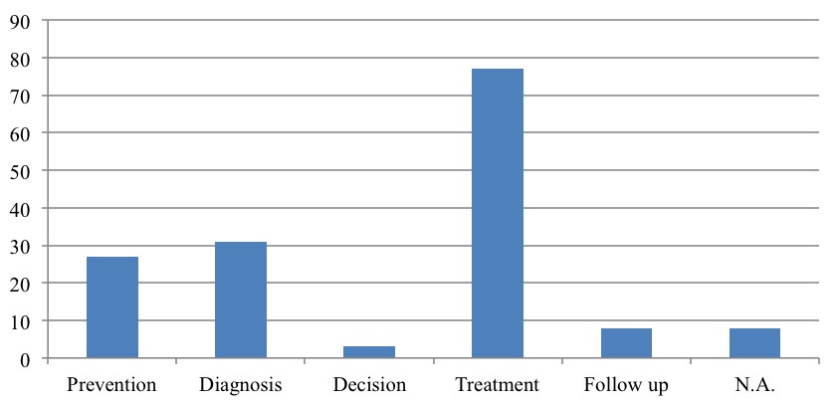
Papers on the different phases of the health care process.

If we look specifically at individual health care processes, we found that mHealth can play a role in all stages of the care process, namely prevention, diagnosis, decision, treatment, and follow-up. However, evidence focuses only on specific phases and most papers suggest a use for treatment purposes [14,35,36,39]. This is because the treatment phase includes all self-management activities carried out by patients [38,46]. Some papers also suggest a role for diagnosis [21,30,47] and a few papers look at prevention [37,41]. A minority of papers look at follow-up [8,48] and there is limited evidence on using mHealth for decision making [49,50] (Figure 7). Consequently, there is scant evidence about using mHealth for integrated care processes or to support new models of care.

**Figure 8:**
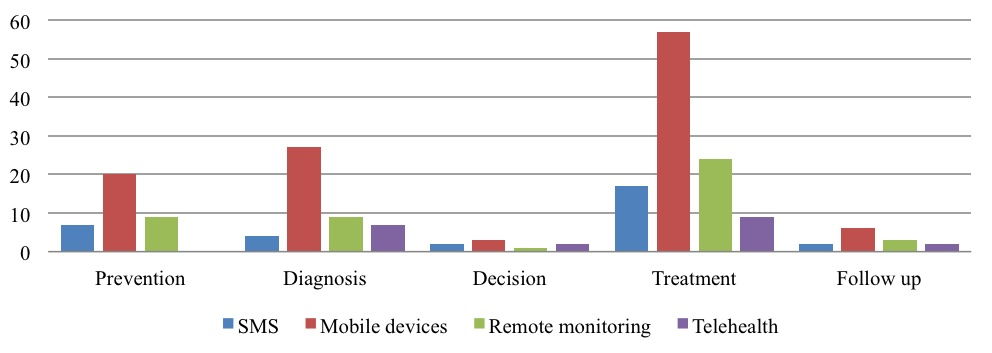
Mobile technologies in health care process phases.

Analyzing the health care process in more detail, we observed the different types of technology used in the phases of the care delivery process (Figure 8). The distribution of mobile technologies used in the different phases of the care process reflects the distribution shown in Figure 4. In particular, we noted the predominant use of smartphones and apps [29,36,37,50] in all phases followed by remote monitoring devices [20,43], even if fewer papers reported this (Figure 5).

On the contrary, less marked differences were observed for the decision [49] and follow-up [8] phases. Since mobile devices like smartphones are used predominantly for self-management activities, the treatment phase features a high use of this type of technology [35,46]. Remote monitoring was the second-most prevailing technology we observed, even if there is a remarkable difference compared to the use of mobile devices. Remote monitoring devices also seem to be used mainly for treatment [46]. Looking at the decision [49] and prevention [16] phases, we observed fewer differences in use, probably because a limited number of papers looked at these stages of the care process.

Finally, looking at how the implementation of mobile health systems is paid for and who pays for it, we noted the whole range of solutions, even if literature does not currently examine this aspect adequately. There are projects [7,37] built entirely in-house, others that are funded by the European Community [16,45], and others requiring both public and private institutions [25] to contribute.

Based on our analysis we found interesting results concerning other types of chronic diseases, such as diabetes, which is mentioned in 18 of our 106 papers. Together with cancer, stroke, and chronic obstructive pulmonary disease (COPD), diabetes is on the list of the major chronic diseases responsible for more than 60% of deaths in the world [51]. The mobile device is the main technology adopted but text messaging and remote monitoring devices are also used. Larsen [39] showed how a mobile phone with a pre-configured app and a Bluetooth-enabled blood glucose meter supported the optimization of insulin dosage, improving control of blood sugar levels.

### Focus on Cancer Supportive Care

**Figure 9:**
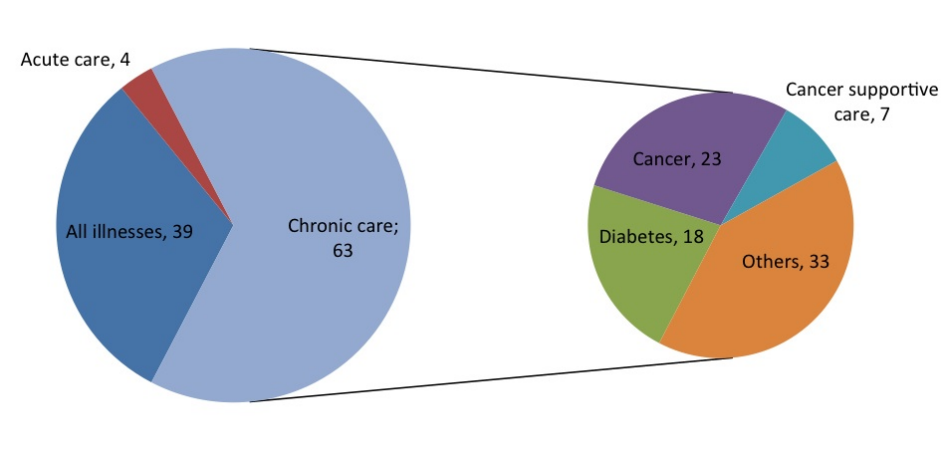
Analyzed diseases.

Nevertheless, cancer supportive care remains the main focus of our research. We found that the role of mHealth in cancer supportive care does not seem to be sufficiently or adequately investigated in literature. Even if our search strategy aimed to look at papers related to mHealth in cancer supportive care, our actual results show that only 59.4% (63/106) of the papers focused specifically on chronic diseases, a category including cancer and cancer supportive care (Figure 9).

Two researchers subsequently screened the records fulfilling our eligibility criteria (n=63) and excluded those that were not pertinent. With regard to the exclusion criteria, the researchers considered certain records as not pertinent after reading the articles themselves; those that did not match the definitions of our streams of research were excluded. This section therefore concentrates on 30 references regarding cancer and cancer supportive care.

**Figure 10:**
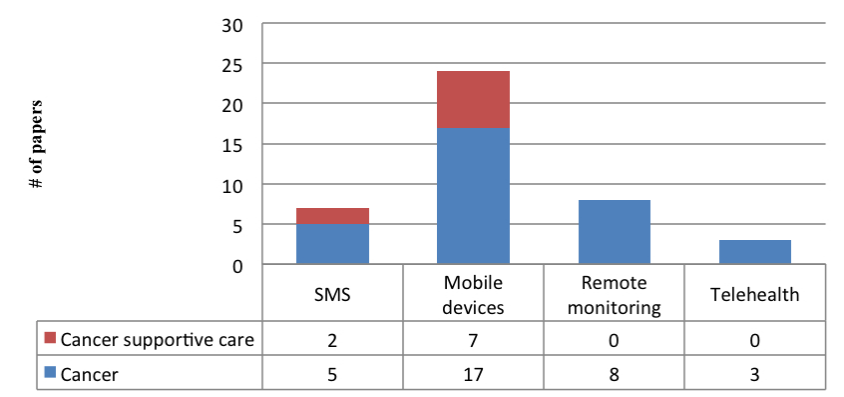
Mobile technologies and diseases.

Focusing on cancer supportive care, mobile devices and apps are the main technology adopted, but text messaging is also used. This may be related to the fact that cancer supportive care revolves mainly around the management of symptoms, and mobile devices and apps are the type of technology used for the most part in this type of activity (Figure 10).

Jaatun [40] analyzed the case of an iPad-based pain assessment tool, developed with a user-centered design, compared to paper-based and conventional laptop-based tools.

We also investigated the Advanced Symptom Management System (ASyMSA) proposed by Kearney [52]. This system requires patients to fill in an electronic symptoms questionnaire and then immediately sends them written feedback via the mobile phone interface, including tailored self-care advice related directly to their symptoms. Patients use a handheld computer to record and send in daily symptom reports to the cancer care center and receive instant, tailored symptoms management advice based on a two-treatment cycle [52]. Finally, Mooney [53] analyzed a daily telephone-linked care (TLC) system for a single cycle of chemotherapy and reporting on seven common chemotherapy-related symptoms. Using selected symptom data, symptoms that met a preset severity threshold generated a fax notification of the patient’s symptom pattern sent to their physician.

Since few papers examined cancer supportive care and focused mainly on self-management, we looked at cancer care in more general terms. Again, smartphones and mobile apps are the most commonly used technology (Figure 10) [54].

**Figure 11:**
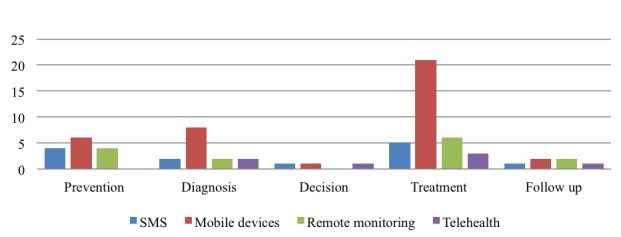
Mobile technologies in the phase of health care processes: focus on cancer care.

When we looked at the health care process in detail, we observed the different types of technology used in the phases of the cancer care delivery process (Figure 11) and failed to find any specific differences from the results presented in Figure 8. We again noted a prevailing focus on treatment activities based on mobile devices, with the decision and follow-up phases being rarely analyzed.

Finally, looking at the location where the pilot and case studies were conducted, we noticed a sharp prevalence of studies conducted in cancer centers [35,39,52], although there is limited evidence.

## Discussion

### Principal Findings

Our analysis of the use of mHealth in cancer supportive care revealed that few papers focus on this particular field, despite the fact that cancer affects more and more people every day. Looking at cancer care in general, we found that the use of mHealth is limited to certain technologies and certain phases of the care process. In particular, we observed that the main technology used consists of mobile devices, and the most explored stage in the health care process is the treatment phase.

The prevalent use of smartphones and remote monitoring devices indicates that mHealth typically supports the automation of processes, focusing on structured activities, such as the automatic transmission of a patient’s vital signs, and in some cases on semi-structured activities. Consequently, it seems that remote monitoring devices are used mainly in the treatment phase, even if this type of technology could also be used in the follow-up phase.

Unstructured activities, mainly consisting of human-executed activities, are supported by mobile technology to a lesser degree, as we found for telehealth and remote surgery.

Regarding the stages in the health care process, not all of them feel the impact of mHealth. The use of mobile technologies concentrates on the treatment phase, mainly because of the extensive use for self-management activities. On the other hand, the decision, prevention, and follow-up phases are hardly affected by the use of mobile technologies, both for cancer and diabetes cases, but this can be explained by the fact they are also the least analyzed by selected literature.

In introducing mHealth, it should be remembered that some uses of mHealth have limited potential. For instance, productivity and efficiency goals can be met if mHealth is used for data collection or to support structured activities. Goals, such as improved effectiveness, can be met if it is used to support clinical decision making, for example, more prompt decision making with an impact on increasing the life expectancy of a cancer patient.

Consequently, if the objective of mHealth is to contribute to an organization’s efficiency, in terms of cost cutting and time saving, it can be used to support data collection in a reliable, accurate, and validated way. If the objective is to reduce the length of hospital stays or re-hospitalization rates, it should be embedded into care process activities. Along with productivity and efficiency goals, mHealth can also make a contribution to the outcomes and results achieved, mainly related to the patient’s perspective and the benefits they can achieve by means of mobile technologies. The concept of the quality of life thus gains importance and is mainly related to improvements to a patient’s health and behavior.

### Conclusions

The results of our analysis show that mHealth is a broad concept that can have several uses and different degrees of pervasiveness in the health care process. Nowadays, mHealth is used in various fields related to chronic diseases, such as diabetes and cancer. However, it is still underutilized in cancer supportive care compared to its potential contribution and mHealth will only be able to support new models of care if it has a high degree of pervasiveness and a wider range of applications. Since mHealth is used for limited purposes and only in some stages of the care process, it is unlikely that it will make a real contribution in achieving new models of care.

This underutilization may depend on many issues, including environmental, regulatory, technological, organizational, and opportunistic questions [55]. It may also depend on the vision shared by health care providers with regard to the actual potential of mHealth and other technologies if applied to care processes, and the strategy they put in place in order to move in that direction. This underuse of mHealth could be due to a failure to embed it into broader information systems [56].

We suggest that we need a better understanding of the reasons for introducing mHealth: if the aim is to achieve integrated models of care, using mHealth should not be limited to certain activities or phases of the health care process. Together with other technologies, mHealth can really make a difference by enhancing performance [57,58] and improving the quality of life of cancer patients. However, this implies adequate use as part of the care process, along with adequate vision, systematic and consistent use, and alignment with the actual objectives that organizations, decision makers, and stakeholders [59,60] really want to achieve with the use of mHealth and any other technologies.

## Abbreviations

SMS: short message service
